# Pain Medicine Education in Emergency Medicine Residency Programs

**DOI:** 10.7759/cureus.37572

**Published:** 2023-04-14

**Authors:** Sunny Bang, Bu M Kong, Oluseyi Obadeyi, Sharmin Kalam, Michael J Kiemeney, Ellen Reibling

**Affiliations:** 1 Department of Emergency Medicine, Loma Linda University Medical Center, Loma Linda, USA

**Keywords:** emergency medicine training, teaching in emergency medicine, graduate medical education, pain medicine, emergency pain management

## Abstract

Background

Pain is a common complaint in the emergency department (ED), yet there is a lack of robust pain curricula in emergency medicine (EM) residency programs. In this study, we investigated pain education in EM residencies and various factors related to educational development.

Methodology

This was a prospective study collecting online survey results sent to Program Directors, Associate Program Directors, and Assistant Program Directors of EM residencies in the United States. Descriptive analyses with nonparametric tests were performed to investigate relationships between these factors, including educational hours, level of educational collaboration with pain medicine specialists, and multimodal therapy utilization.

Results

The overall individual response rate was 39.8% (252 out of 634 potential respondents), representing 164 out of 220 identified EM residencies with 110 (50%) Program Directors responding. Traditional classroom lectures were the most common modality for the delivery of pain medicine content. EM textbooks were the most common resource utilized for curriculum development. An average of 5.7 hours per year was devoted to pain education. Up to 46.8% of respondents reported poor or absent educational collaboration with pain medicine specialists. Greater collaboration levels were associated with greater hours devoted to pain education (p = 0.01), perceived resident interest in acute and chronic pain management education (p < 0.001), and resident utilization of regional anesthesia (p = <0.01). Faculty and resident interest in acute and chronic pain management education were similar to each other and high on the Likert scale, with higher scores correlating to greater hours devoted to pain education (p = 0.02 and 0.01, respectively). Faculty expertise in pain medicine was rated the most important factor in improving pain education in their programs.

Conclusions

Pain education is a necessity for residents to adequately treat pain in the ED, but remains challenging and undervalued. Faculty expertise was identified as a factor limiting pain education among EM residents. Collaboration with pain medicine specialists and recruitment of EM faculty with expertise in pain medicine are ways to improve pain education of EM residents.

## Introduction

Pain is the most common complaint in the emergency department (ED) [1,2]. With over 135 million visits to EDs in the United States per year [3], pain-related visits can be as high as 78% (adult and pediatric), with up to 40% of them presenting with underlying chronic pain processes [2,4]. Unrelieved pain negatively impacts patients in a variety of ways physically and socially. Acutely, physiologic responses can include increased blood pressure, muscle spasms, decreased immune function, and distressing cognitive impairment [5]. Persistent pain heightens the risk of developing chronic pain, which can significantly alter a patient’s quality of life and negatively affect the management of other chronic diseases [6,7]. It can also increase susceptibility to developing neuropsychiatric complications [6]. Consequently, this requires emergency medicine (EM) physicians to be versed in pain management. Pain reduction in the ED decreases patients’ stress levels, improves rapport and satisfaction with physicians, and contributes to earlier mobilization with a return to activities of daily living [8,9]. It may also be associated with a decrease in return visits to the ED [10]. However, several studies have reported the undertreatment of pain, otherwise known as oligoanalgesia, varying from older patients and even in relation to race and ethnicity [1,11-13]. This is followed by a notable lack of awareness in addressing the problems of chronic pain patients who tend to not have established pain specialty care, leading to frequent utilization of ED services while reporting dissatisfaction with their ED pain management [4,14,15]. Chronic pain patients are also estimated to make up 20% of the general US population, which further worsens these issues [4,14-16].

Overall, there is an important need for EM physicians to develop competencies in both acute and acute-on-chronic pain management in the ED, as they are constantly treating a clinically diverse group of patients (chronic pain syndromes, substance use disorders, children, elderly, etc.). Education is an effective approach to facilitate these needs [17-19]. Yet, medical school education on pain medicine is currently inadequate and highly variable before residency, as it is not a required clinical topic by the Liaison Council of Medical Education [20,21]. Although the 2019 Model of the Clinical Practice of Emergency Medicine lists pain management as a core learning topic during residency, there are no recommendations regarding how the curriculum should be taught and what clinical principles to generally cover [22]. In response, an EM pain education curriculum was defined, which recommended the creation of pain management modules mixed with short didactics and cases, incorporating pain management concepts into current EM milestones, safe prescribing measures, and communication skills when interacting with patients in significant pain [23]. Furthermore, no described curriculum exists for ED chronic pain management. The degree to which programs are incorporating pain education into their curriculum is unknown. Our study attempts to reveal the current status of general pain education within EM residency programs, as well as highlight factors that may contribute to limiting pain education development.

## Materials and methods

Study design

This is a prospective, cross-sectional study that uses a closed survey to collect data from EM residencies in the United States. All protocols, including the survey, were reviewed and approved by the Loma Linda University Health Institutional Review Board. Survey questions were developed in conjunction with core EM and pain faculty at our institution and two nearby institutions that also have their own EM residency programs. Our core EM faculty consisted of individuals trained in medical education fellowships or who have held an Associate or Assistant Program Director position in the past. The survey was piloted among former EM Program and Associate/Assistant Program Directors and revised based on the validity of the questions and error testing. The final survey included questions focused on general pain education, such as educational modalities, resources for curriculum development, resident evaluation, and professional relationships with pain medicine specialists (who have undergone training in Pain Medicine fellowship programs and/or board-certified in Pain Medicine). Participants could submit the survey without answering every single question to allow for increased response rates.

Study setting and population

The study population consisted of Program Directors (PD), Associate Program Directors (APD), and Assistant Program Directors (aPD) of EM residency programs in the United States. At the time of the study, there were 269 EM programs accredited by the Accreditation Council of Graduate Medical Education based on their online database [24]. However, several of these programs were recruiting their first class to start in July 2020 or 2021. As a result, we excluded programs without third-year residents starting in July 2020, as they were newly established without considerable time spent training residents or having a fully developed curriculum on pain management. This resulted in a total of 222 EM programs available as potential programs to contact for participants. The names and contact information were obtained from reviewing the information listed on their residency websites. We utilized our EM residency email mailing list to assist with this process as well. When data were missing or incomplete, we attempted to contact program coordinators to either verify PD, APD, and aPD information or help forward the survey to their email addresses for participation. Two EM programs stated their policy was to not participate in survey studies, resulting in a total of 220 EM programs. We were unable to acquire the e-mail addresses of 34 participants after attempting contact with their program coordinators. This resulted in a list of 220 PDs, 227 APDs, and 187 aPDs, totaling 634 potential participants.

Study protocol

The survey was created and distributed electronically using Qualtrics (Qualtrics, Seattle, WA, USA). The finalized survey is provided in Appendix 1. Personalized links were distributed to participants by email. This allowed for the investigators to determine who completed the survey and collect their faculty position and program. This also allowed for measurements of multiple responses from the same program. However, anonymity was still maintained, as no location data, IP address, or email was attached to these responses, so the data could not be connected back to the respondents. An online consent form was displayed to the participants before proceeding to the survey questions. Submission of the survey served as consent for participation. Participants were not compensated for participating.

Initially, the first round of surveys was sent in December 2020, but after evaluating the current state of the COVID-19 pandemic and related stressors, the decision was made by research personnel to pause the study and resume at the end of March 2021. The list of possible participants was not re-evaluated, as this was only a four-month time difference and deemed to have a lower likelihood of faculty changes in the middle of the academic year. Furthermore, the survey asked for their faculty position at the beginning. A reminder email was sent to participants who had not completed the survey every three to four weeks until the first week of October 2021. Survey results were recorded and stored on a secure departmental computer, which included the number of formal educational hours, educational formats, resources, Likert scale scores, and availability/involvement of pain medicine specialists. The Checklist for Reporting Results of Internet E-Surveys (CHERRIES) was utilized to guide reporting in this study and is provided in Appendix 2 [25].

Data analysis

Descriptive analyses were used for participant demographics. Descriptive statistics were also performed on Likert-type questions with assigned modes and visual representation of the frequencies for each choice. When a respondent gave a range as an answer (such as four to six hours), the average was taken between the range. Statistical analyses were performed to investigate the relationship between the time devoted to pain management education at an individual residency program and several variables, such as the geographic location of the program and pain medicine specialist involvement in education. We analyzed the effects of educational collaboration with pain medicine specialists on perceived resident interest in pain management education and utilization of regional anesthesia, opioid-sparing analgesics, and nonpharmacologic adjuncts (modalities that are not pharmacologic in nature, such as ice packs, bracing, early mobilization, educational advice, and splinting) in the clinical environment. Opinions toward pain education improvement were analyzed for PDs, APDs, and aPDs. For these outcomes, median and quartile responses at 25% and 75% were utilized for the distribution of these variables. Nonparametric tests were used because the data were not expected to be distributed normally and typically skewed. Kruskal-Wallis tests were performed to compare median Likert scores with follow-up Wilcoxon two-sample tests for confirmatory analysis where differences were statistically significant. A two-sided p-value <0.05 was considered statistically significant. A chi-square analysis (p < 0.05) was used to assess for categorical differences. When there were multiple responses from the same institution, participants were analyzed by their position (PD, APD, and aPD) as opposed to a group in comparison to groups of other institutions. Due to the anonymous nature of our survey design, we were not able to calculate an agreement of responses from participants of the same institution. For the last question of the survey, respondents were asked to rank five specific factors in order of importance (1 being most important) in improving pain medicine education in their EM residency program. These factors were faculty expertise and experience, institutional support, resident interest in pain management, faculty interest in pain management, and collaboration with pain medicine specialists. Free responses at the end of the survey regarding pain education in residency programs were reviewed and organized into the following three categories based on similarly recurring themes: innovative approaches, ideas to improve pain education, and barriers to pain management in the ED.

## Results

Characteristics of study subjects

Of the 634 potential participants contacted, the overall response rate was 39.8%, with 252 individuals responding. Of the 220 EM residency programs identified, 164 (74.5%) EM programs responded to the corresponding survey, 138 (84.1%) of which were three-year programs while 26 (15.8%) were four-year EM programs. Overall, 50% (n = 110) of PDs, 39.6% (n = 90) of APDs, and 27.8% (n = 52) of aPDs responded (Table 1). The majority of the respondents were from the Southeast (27.9%), Midwest (25.9%), and Northeast (28.6%) regions, with the least from the Southwest (3.2%) and West (12.7%). In addition, 85% of respondents reported having access to pain medicine specialists for educational collaborative efforts. Only 70 (42%) programs had an institution-sponsored pain medicine fellowship.

**Table 1:** Selected demographics of respondents. Subtotal percentages may not add up to 100% because of rounding. All = all respondents; PD = program director; APD = associate program director; aPD = assistant program director

Variable	% (All)	% (PD)	% (APD)	% (aPD)
Gender				
Male	59.9 (n = 151)	28.2 (n = 71)	20.6 (n = 52)	11.1 (n = 28)
Female	37.7 (n = 95)	15.1 (n = 38)	13.5 (n = 34)	9.1 (n = 23)
Unknown	2.4 (n = 6)	0.4 (n = 1)	1.2 (n = 4)	0.4 (n = 1)
Total	100.0 (n = 252)	43.7 (n = 110)	35.3 (n = 90)	20.6 (n = 52)
Geographic breakdown (%)				
West	12.7			
Southwest	3.2			
Southeast	27.9			
Midwest	25.9			
Northeast	28.6			
Unknown	2.0			
Training location (%)				
Urban	81.7			
Suburban	15.1			
Rural	1.2			
Unknown	2.0			

Teaching modalities and educational resources

**Figure 1:**
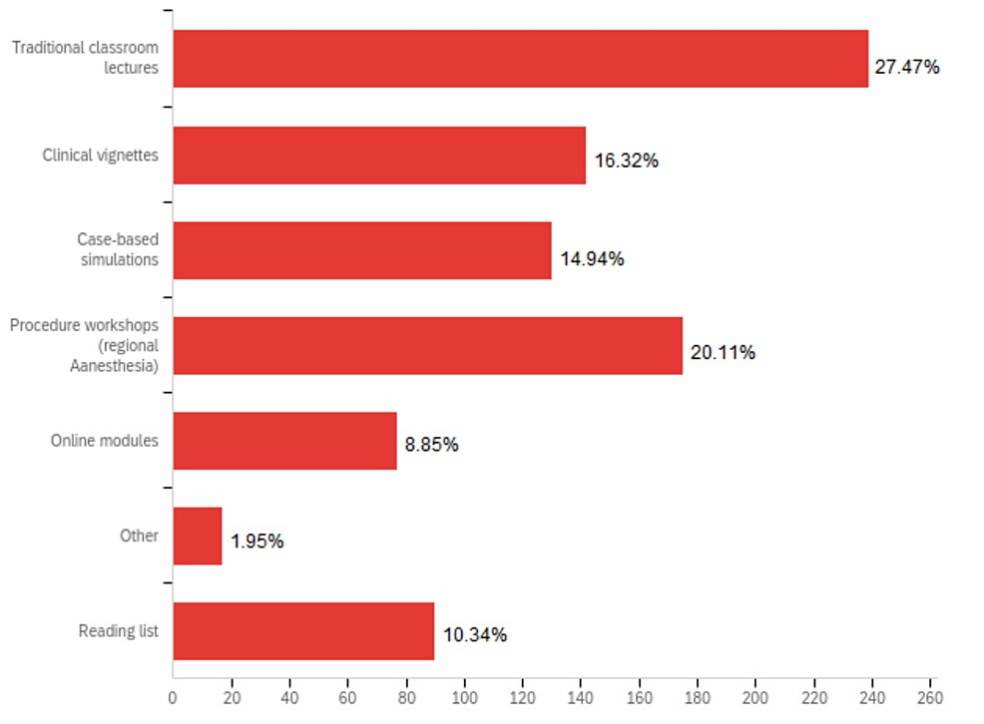
Teaching modalities utilized to deliver pain management content. Survey question: What teaching modalities do you use to deliver pain management content? Content examples include addressing chronic pain, opioid prescribing risks, pharmacology, regional anesthesia, etc. (please select all that apply). Note: The above figure was generated thanks to the Qualtrics platform.

**Figure 2:**
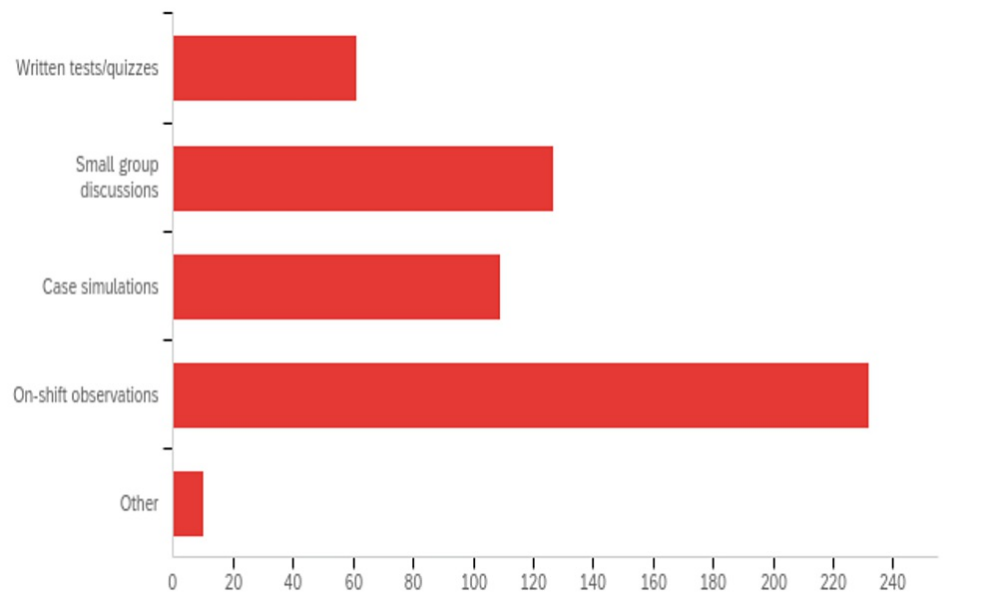
Methods used to evaluate resident competency in pain management knowledge and skills. Survey question: What methods do you use to specifically evaluate your residents’ pain management knowledge and skills? (please select all that apply). Note: The above figure was generated thanks to the Qualtrics platform.

When respondents were asked to select modalities used to teach pain management in their program, most respondents (94.8%) selected traditional classroom lectures (Figure 1) in their institution. The second most common way to deliver content related to pain management topics was through procedure workshops (69.4% of respondents). Other common modalities of teaching mentioned that were not part of the available options included small group discussions, elective rotations, and residency emails updating pain medication usage. The most common resources utilized for pain education development were emergency medicine textbooks (76.6%) and Free Open Access Medical Education (FOAMed) (71.8%). Residents’ knowledge and skills in pain medicine were mainly assessed when their performances were observed during clinical shifts (92.1%) (Figure 2). Small group discussions and case simulations were the next most common method for evaluation, accounting for 50.4% and 43.3%, respectively. Few respondents mentioned that pain management was not formally assessed in their programs.

Exposure to pain management education and ED interventions

Participants were asked to estimate the number of formal hours (based on the teaching modalities) devoted to pain education in an average year. An average of 5.7 formal educational hours per year were devoted to pain education with a 95% confidence interval (CI) of (5.0, 6.4), while some respondents reported that they spent zero formal hours. Furthermore, 63% of respondents stated that their residents have the opportunity to rotate in pain medicine-related electives (regional anesthesia, chronic pain service, acute pain service, interventional pain management, etc.), while 37% did not have such access at their institution. The number of programs allowing for these opportunities was not calculated due to the anonymity of the survey. There was no significant difference with the length of the EM program (H = 0.48, p = 0.49), geographic location (χ^2^ = 89.37, p = 0.50), and opportunity to rotate in pain medicine-related electives (χ^2^ = 27.35, p = 0.07) in relation to the number of formal hours devoted to pain education.

**Figure 3:**
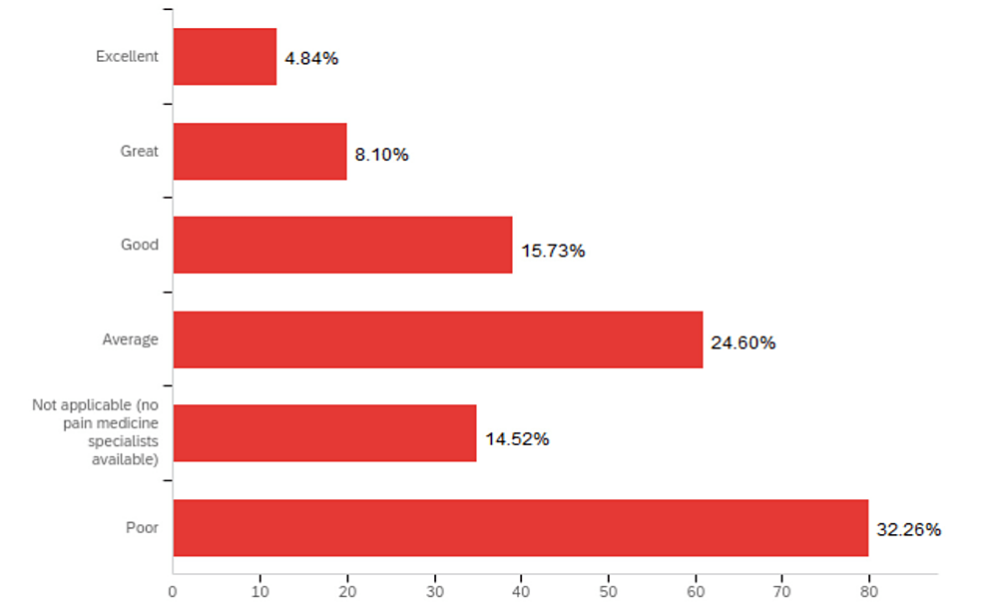
Level of educational collaboration with pain specialists. Survey question: What is the level of educational collaboration (didactics, procedure workshops, consults, etc.) with pain medicine specialists at your program? Note: The above figure was generated thanks to the Qualtrics platform.

Illustrated in Figure 3, 46.8% of respondents stated their program had either poor (32.26%) or absent (14.52%) educational collaboration (didactics, procedure workshops, consults, etc.) with pain medicine specialists, while only 4.84% of respondents reported having an excellent level of educational collaboration with pain medicine specialists at their program. Although EM programs from the West and Southeast reported greater levels of educational collaboration with pain medicine specialists, there was no statistically significant difference among the geographic regions (χ^2^ = 7.67, p = 0.18). However, greater levels of educational collaboration with pain medicine specialists were associated with a higher number of formal hours devoted to pain education (χ^2^ = 14.72, p = 0.01).

Respondents were also asked to select how often their residents utilize components of multimodal analgesic therapy, including regional anesthesia, opioid-sparing analgesics, and nonpharmacologic adjuncts, in their patient care (Table 2). Notably, 38.8% of respondents reported their residents do not often utilize regional anesthesia. Up to 90.9% reported their residents always or often utilize opioid-sparing analgesics. Overall, 35.5% reported their residents do not often utilize nonpharmacologic adjuncts. Opioid-sparing analgesics were more often utilized (mean = 4.1, SD = 1.0) than regional anesthesia (mean = 3.0, SD = 1.0) and nonpharmacologic adjuncts (mean = 3.2, SD = 0.6). However, EM programs with greater levels of educational collaboration with pain medicine specialists reported residents with higher utilization rates of regional anesthesia (χ^2^ = 15.6, p < 0.01). There were no statistically significant differences in the utilization of opioid-sparing analgesics (χ^2^ = 1.34 p = 0.71) and nonpharmacologic adjuncts (χ^2^ = 7.58, p = 0.1) in relation to levels of educational collaboration with pain specialists.

**Table 2:** Utilization of regional anesthesia, opioid-sparing analgesics, and nonpharmacologic adjuncts. Survey question: In the ED, our residents commonly utilize components of multimodal therapy listed below. SD = standard deviation; ED = emergency department; 1 = Never; 2 = Not often; 3 = About half the time; 4 = Often; and 5 = Always

	Always	Often	About half the time	Not often	Never	Mean Likert score (SD)
Regional anesthesia	5.0%	34.3%	20.3%	38.8%	1.7%	3.0 (1.0)
Opioid-sparing analgesics	25.1%	65.8%	7.4%	1.6%	0.0%	4.1 (0.6)
Nonpharmacologic adjuncts	8.7%	35.6%	19.0%	35.5%	0.8%	3.2 (1.0)

Interest in pain management

A five-point Likert scale was used to assess the general interest in acute and chronic pain management education among residents and faculty. Overall, 12.8% and 31.4% of respondents indicated that residents were extremely interested and very interested in learning about pain management, respectively. Notably, greater resident interest was associated with higher educational hours devoted to pain education (χ^2^ = 9.72, p = 0.02). Resident interest was also correlated with greater levels of educational collaboration with pain specialists (H = 23.64, p < 0.001). There was no correlation between resident interest and opportunities to rotate in pain medicine-related electives (χ^2^ = 2.10, p = 0.55).

Faculty interest in pain management education was similar to that of residents. Overall, 8.3% and 28.9% of respondents said that faculty members were extremely interested and very interested in pain management, respectively. Moreover, greater faculty interest was associated with higher educational hours devoted to pain education (χ^2^ = 13.19, p = 0.01).

Perceived factors in improving pain education

Faculty expertise and experience were selected as the most important factor, followed by faculty/resident interest, collaboration with pain medicine specialists, and, lastly, institutional support. There were no significant contrasts between faculty positions (PD, APD, and aPD) in relation to the ranking of these five factors.

General comments about pain education

**Figure 4:**
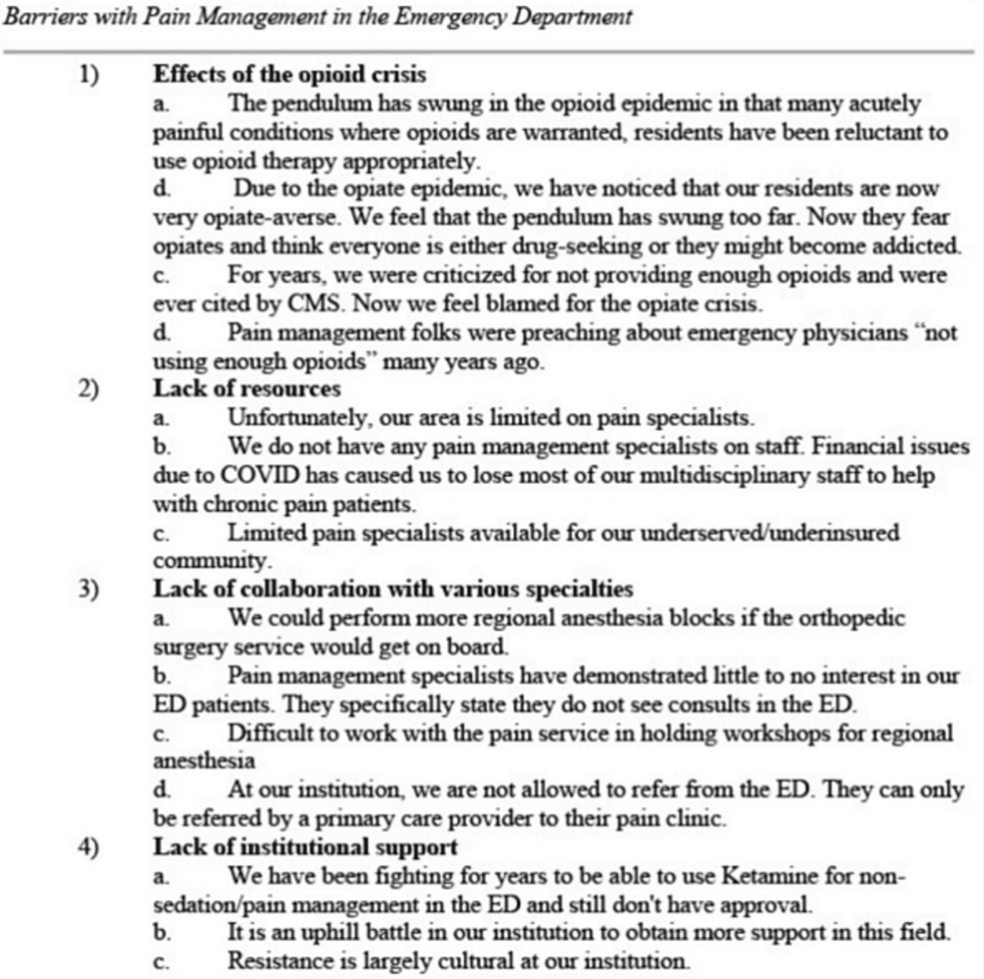
General thoughts about pain education in emergency medicine residency programs, part 1. ED = emergency department, CMS = Center for Medicare and Medicaid Services

**Figure 5:**
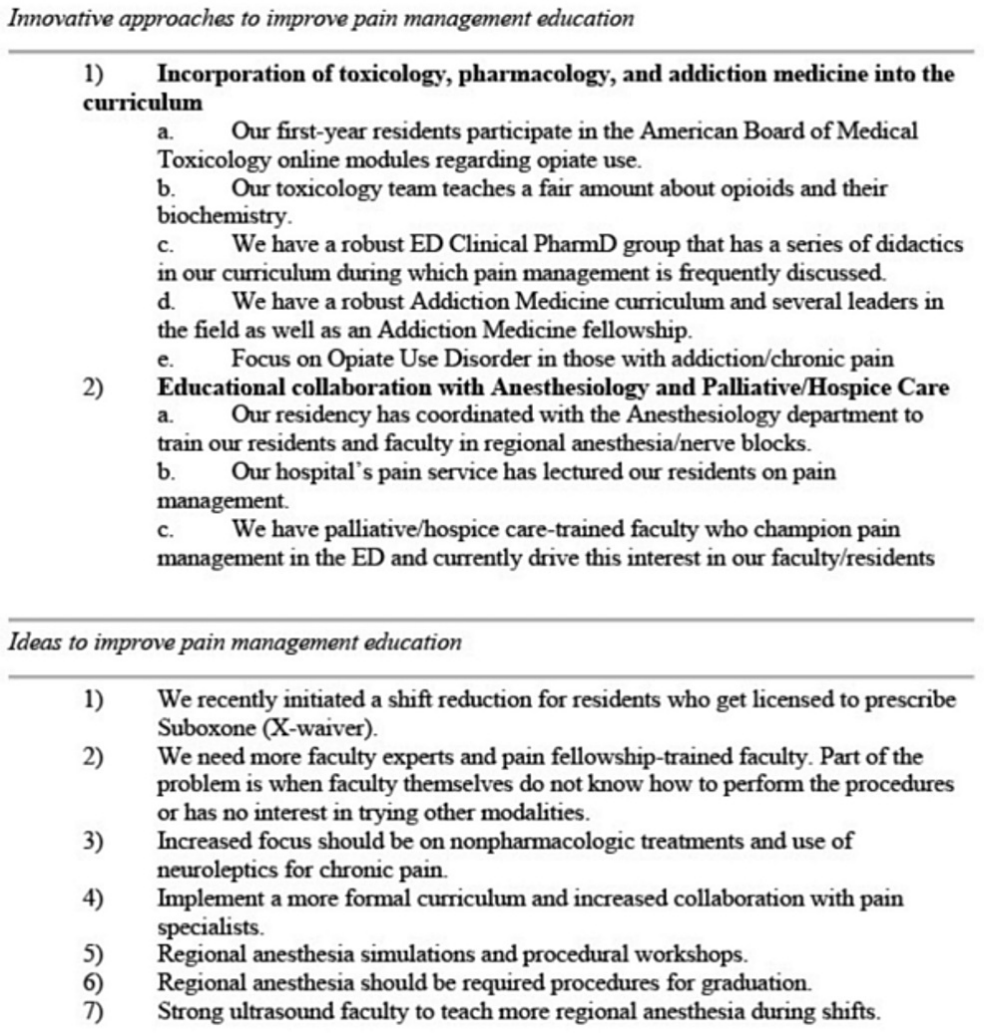
General thoughts about pain education in emergency medicine residency programs, part 2. ED = emergency department

Free responses regarding pain education were organized (Figures 4, 5) into three categories, namely, multiple barriers that emergency departments face with pain management, innovative approaches that are already implemented by other programs, and new ideas that will improve pain education in EM residencies. Innovative approaches largely centered around collaborating with multidisciplinary experts at their respective institutions to educate their residents. Several comments highlighted the importance of faculty expertise, especially in regional anesthesia, to lead the charge in pain management in the ED. Lastly, many felt there was not enough collaboration with pain medicine specialists or institutional support, such as orthopedic surgery not allowing for ED providers to perform regional anesthesia on their patients or institutional approval for the use of ketamine for pain.

## Discussion

Several studies have highlighted the inadequacy of pain management in EDs [1,4,11-16]. As a result, pain medicine is an area of growing importance in EM, making it necessary for EM physicians to be skilled in its practice. There are several studies surveying patient satisfaction with respect to pain control in the ED with reported oligoanalgesia, but none that we are aware of assess pain education in EM residencies. Our study is the first to evaluate various aspects of pain education in EM residencies across the United States from program leadership.

The most commonly used modality to teach pain education was traditional classroom lectures. However, prior research has shown that lectures and classroom teaching have poor retention rates, with more programs switching to small group and case-based discussions [26,27]. Instead, active learning has been shown to be a more effective educational technique. Interactive workshops and courses in controlling pain in ED patients improved after training EM residents [19]. This is in line with consensus-based recommendations for a broad and modular pain management curriculum in EM programs [23]. When evaluating resident competency in pain medicine knowledge and skills, tests and quizzes are not able to fully evaluate the procedural technique required for modalities, such as regional anesthesia. This is one possible explanation for why residency programs more often use case simulations and on-shift observations to evaluate competency in pain management. Fortunately, multiple studies have demonstrated that hands-on educational interventions are effective [26,27]

Prior studies have revealed a lack of faculty interest as a perceived barrier in the delivery of educational training in graduate medical education programs [28,29] Therefore, it is reassuring and important to note that most faculty and residents were interested in pain education. The greater their interests were, the greater the number of formal educational hours and collaborations with pain medicine specialists. Nonetheless, increased interest in an educational topic does not necessarily translate to better knowledge and outcomes, as other barriers may impede them. For instance, faculty expertise was still rated as the most significant barrier to improving pain education, as residents learn clinically and procedurally from core faculty during academic sessions and ED shifts. Several comments at the end of the survey echoed the same sentiments, such as stating that residents are unable to be proficient in regional anesthesia due to the lack of supervising faculty members. Thus, our study suggests a need for recruiting EM physicians who are experts in pain medicine or fellowship-trained in pain medicine into their faculty to help with pain medicine curriculum development and clinical teaching on shifts.

Considering all the core competencies and procedural skills that need to be covered during training, it is unsurprising that only 5.7 formal hours in an average year are dedicated to pain education. This can be interpreted as one hour of pain medicine concepts every other month or a full conference day per year. Although most of our respondents were leaders of three-year programs, which can experience more time constraints in teaching all the required EM core competencies, we did not anticipate that our data analysis would show no differences between three and four-year EM programs in the amount of time dedicated to pain medicine education. However, collaborating with pain medicine specialists may increase the average number of formal hours devoted to pain management education, as our data suggested that levels of collaboration were directly correlated with the number of hours for education. Unfortunately, most programs had poor levels of collaboration and even no pain medicine specialists in a few programs. This is in the context of only 111 pain medicine fellowships available in the United States, with only 92 having a coexisting EM residency program. Lastly, 63% of respondents stated their program has ample opportunities to rotate in pain medicine electives. Still, the number of programs that have these opportunities is lower due to various respondents being from the same program. Furthermore, this is only fruitful if the residents themselves choose to utilize their elective time for this purpose.

Lastly, regional anesthesia utilization among residents was noted to be higher in those with greater levels of collaboration. This is important in the setting that nerve blocks tend to not be standard practice in the ED, despite the reported benefits of better pain control, decreased opiate use, and decreased use of additional analgesia [30]. Respondent comments on our survey echoed this need for more regional anesthesia teaching and implementation and the lack of collaborative efforts. A survey of PDs regarding the barriers to ultrasound-guided regional anesthesia (UGRA) also highlighted the lack of interdepartmental collaboration [31]. The authors suggested addressing this barrier as a potential strategy to develop an EM UGRA curriculum and possibly incorporate residents into UGRA electives with the anesthesia department, just as programs already do for airway training [31]. Therefore, encouraging EM residents to take on pain medicine-related electives may help further their pain management knowledge and skills while strengthening interdepartmental relationships with these services. In all, our findings suggest EM programs should seek out ways within their institution to increase or establish their collaboration with pain medicine specialists to help improve pain management education overall.

Limitations

Limitations of survey research have been documented in the literature [32]. The anonymity of the survey design limits our ability to perform statistical comparisons among respondents in the same institution. We opted for an anonymous survey to encourage respondent participation and to decrease response bias. Although we attempted to reduce response bias, we acknowledge that response bias is intrinsic to any survey design and thus never eliminated in a study. Some of our respondents might have been EM physicians interested in pain medicine education and some of the identified participants who never responded might have been due to a lack of interest in pain education. Recall bias is a consideration, as the estimation from a PD may vary from an APD or aPD. There may be differences between early and late responders of the survey that were not accounted for. Additionally, our survey asked faculty to report their residents’ perceptions of pain management education in their program. It also asked to assess the interest levels of both residents and faculty. As a result, its subjective nature is part of the study’s limitations. Ideally, a concurrent or follow-up survey of residents would also address these topics. The study population was limited to EM residencies in the United States; thus, the findings may not be applicable to other countries. Lastly, what qualified as acute and chronic pain management education was not strictly defined for the purposes of keeping the survey brief. This was left up to the respondents to determine, in a broad and reasonable sense, what could be classified as pain medicine education, but this was also considered a limitation of the study.

The response rate was 39.8%. However, we acknowledge this as a limitation, as this may not be adequate enough to be representative of the population given that responders can differ from nonresponders due to response bias. The study was initially planned to be conducted over a three-month period, but due to the effects of the COVID-19 pandemic and consequential burnout, the study was prolonged to six months of data collection. Future studies need to be conducted to identify if there is a significant difference when patients with a chief complaint of pain receive medical management from EM residents with proficient pain management training (such as a focused curriculum and pain medicine-related electives) compared to limited training. Future studies will also likely benefit from more clearly defining and separating acute and chronic pain management. This also includes the creation and implementation of novel pain curricula and assessing patient-centered outcomes of pain management.

## Conclusions

Pain medicine education is an essential component of EM residencies. Most ED visits are due to conditions related to acute and chronic pain, which makes it a necessity for EM resident physicians to be able to adequately treat pain. Faculty expertise and experience are crucial factors that limit the improvement of pain medicine education within residency programs. Greater collaborative efforts with pain medicine specialists and emergency physician recruitment of experts in pain management or training in pain medicine fellowships to educational positions may enhance pain education in EM residencies.

## Figures and Tables

**Figure 6:**
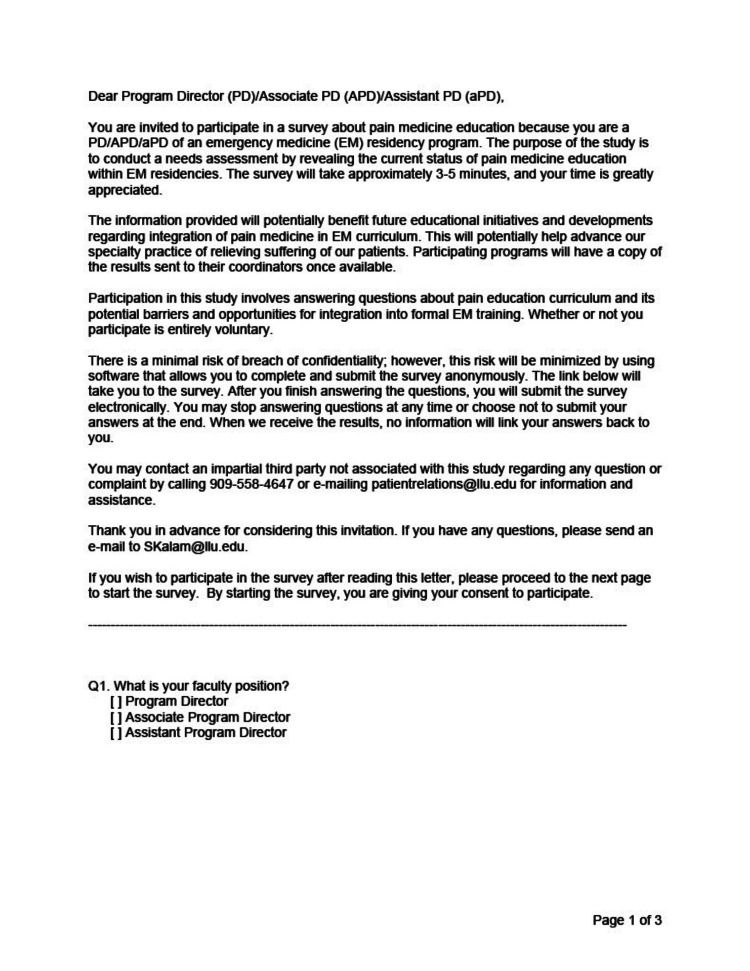
Survey for program directors and associate/assistant program directors, page 1.

**Figure 7:**
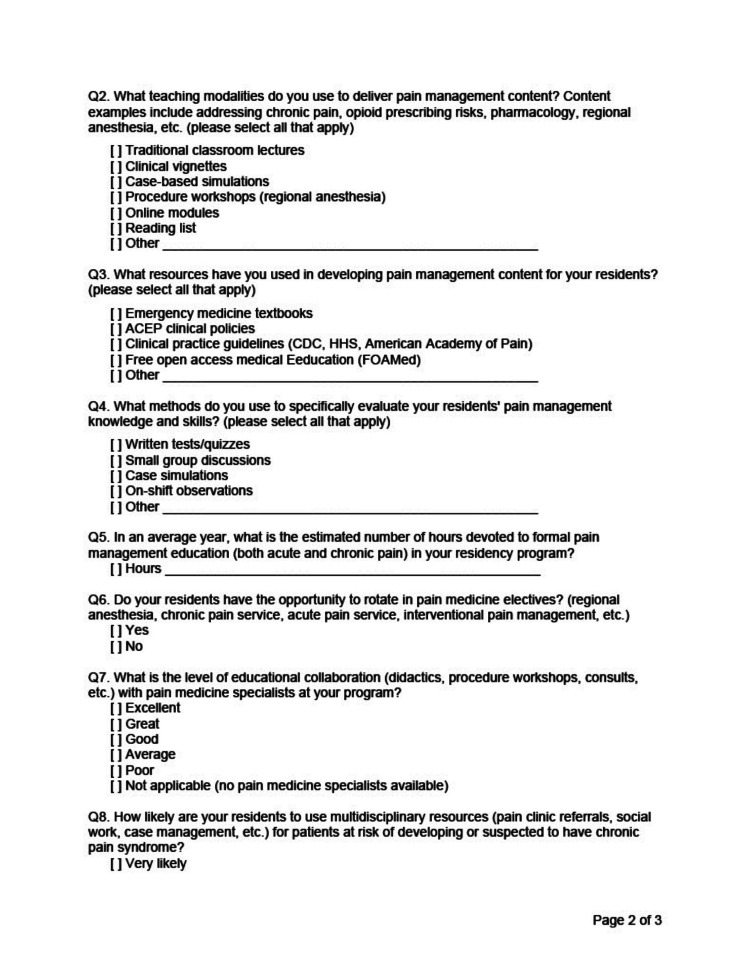
Survey for program directors and associate/assistant program directors, page 2.

**Figure 8:**
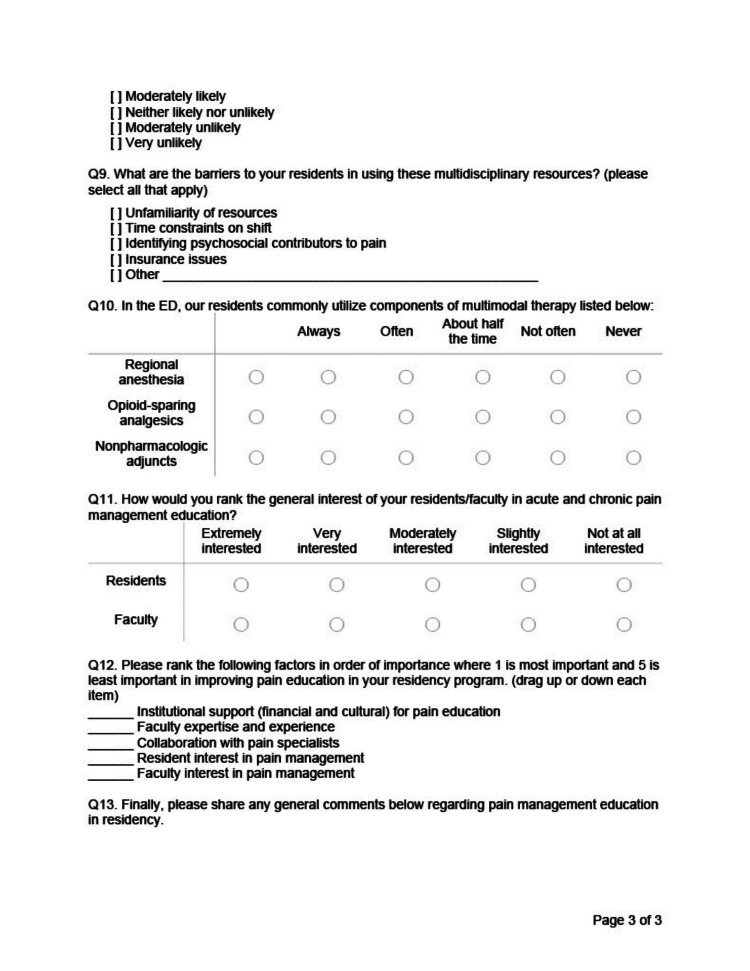
Survey for program directors and associate/assistant program directors, page 3.

## Appendices

Appendix 1

Appendix 2 

**Table 3:** Checklist for Reporting Results of Internet Electronic Surveys (CHERRIES).

Item category	Checklist item	Description
Design	Study design	The target population was 634 PDs, APDs, and aPDs of 220 EM residency programs in the United States
IRB approval and informed consent process	IRB approval	IRB approval was obtained from the Loma Linda University Health Institutional Review Board
	Informed consent	Informed consent for the survey was obtained from all those agreeing to complete the survey by clicking the “Go to Survey” button on the email. All participants were informed in the email that the survey concerned pain medicine education in emergency medicine residencies, it would take approximately 30 minutes to complete, and that all responses were confidential and anonymous
	Data protection	Surveys were saved in an encrypted folder that was saved in password-protected departmental computers
Development and pre-testing	Development and testing	The survey was designed with the help of EM physicians trained in medical education fellowships or who have held an Associate or Assistant Program Director position in the past. The survey was piloted among former EM Program and Associate/Assistant Program Directors and revised based on the validity of the questions and error testing
Recruitment process and description of the sample having access to the questionnaire	Open vs. closed survey	This was a closed survey and only available to the study sample
	Contact mode	Potential participants were emailed an introduction to the study and the corresponding survey
	Advertising the survey	The survey was not advertised
Survey administration	Web/email	The survey was created and distributed electronically using Qualtrics
	Context	Personalized links were distributed to participants by email
	Mandatory/voluntary	Voluntary
	Incentives	Respondents were not incentivized for their participation
	Time/date	Responses were collected between December 2020 and October 2021
	Item randomization	No randomization of items was used
	Adaptive questioning	Adaptive questioning was not used
	Number of items	13
	Number of screens	2
	Completeness check	Completion of all survey items was not mandatory
	Review step	Respondents were unable to change their responses once submitted
Response rates	Unique site visitor	Unique site visitor was handled by Qualtrics, which ensured that respondents only completed the survey once
	View rate	Not applicable given that regardless of view rate, unique site visitor was only allowed to complete the survey only once
	Participation rate	39.80%
Preventing multiple entries from the same individual	Cookies used	Yes
	IP check	Yes
	Log file analysis	Not used
	Registration	Not used
Analysis	Handling of incomplete questionnaires	Only completed questionnaires were included in the final dataset
	Questionnaires with atypical timestamp	No respondents were removed from the survey for completing the items too quickly
	Statistical correction	No statistical correction was utilized
